# Using NanoSIMS coupled with microfluidics to visualize the early stages of coral infection by *Vibrio coralliilyticus*

**DOI:** 10.1186/s12866-018-1173-0

**Published:** 2018-04-20

**Authors:** E. Gibbin, A. Gavish, I. Domart-Coulon, E. Kramarsky-Winter, O. Shapiro, A. Meibom, A. Vardi

**Affiliations:** 1 Laboratory for Biological Geochemistry, School of Architecture, Civil and Environmental Engineering, École Polytechnique Fédérale de Lausanne (EPFL), Lausanne, Switzerland; 2 Weizmann Institute of Science, Rehovot, Israel; 3 Museum National d’Histoire Naturelle, MCAM UMR7245CNRS-MNHN, Paris, France; 4 Volcani Center for Agricultural Research, Rishon LeZion, Israel; 5 Center for Advanced Surface Analysis, Institute of Earth Sciences, University of Lausanne, Lausanne, Switzerland

**Keywords:** Global change, Ocean warming, Coral disease, Stable isotopes, Coral immunity, NanoSIMS

## Abstract

**Background:**

Global warming has triggered an increase in the prevalence and severity of coral disease, yet little is known about coral/pathogen interactions in the early stages of infection. The point of entry of the pathogen and the route that they take once inside the polyp is currently unknown, as is the coral’s capacity to respond to infection. To address these questions, we developed a novel method that combines stable isotope labelling and microfluidics with transmission electron microscopy (TEM) and nanoscale secondary ion mass spectrometry (NanoSIMS), to monitor the infection process between *Pocillopora damicornis* and *Vibrio coralliilyticus* under elevated temperature.

**Results:**

Three coral fragments were inoculated with ^15^N-labeled *V. coralliilyticus* and then fixed at 2.5, 6 and 22 h *post-*inoculation (hpi) according to the virulence of the infection. Correlative TEM/NanoSIMS imaging was subsequently used to visualize the penetration and dispersal of *V. coralliilyticus* and their degradation or secretion products. Most of the *V. coralliilyticus* cells we observed were located in the oral epidermis of the fragment that experienced the most virulent infection (2.5 hpi). In some cases, these bacteria were enclosed within electron dense host-derived intracellular vesicles. ^15^N-enriched pathogen-derived breakdown products were visible in all tissue layers of the coral polyp (oral epidermis, oral gastrodermis, aboral gastrodermis), at all time points, although the relative ^15^N-enrichment depended on the time at which the corals were fixed. Tissues in the mesentery filaments had the highest density of ^15^N-enriched hotspots, suggesting these tissues act as a “collection and digestion” site for pathogenic bacteria. Closer examination of the sub-cellular structures associated with these ^15^N-hotspots revealed these to be host phagosomal and secretory cells/vesicles.

**Conclusions:**

This study provides a novel method for tracking bacterial infection dynamics at the levels of the tissue and single cell and takes the first steps towards understanding the complexities of infection at the microscale, which is a crucial step towards understanding how corals will fare under global warming.

**Electronic supplementary material:**

The online version of this article (10.1186/s12866-018-1173-0) contains supplementary material, which is available to authorized users.

## Background

Coral reefs are highly complex and diverse ecosystems that have considerable ecological and economic value [1]. They thrive in the oligotrophic shallow waters of the tropics because of a highly dynamic and tightly regulated symbiosis that exists between the coral animal, their photosynthetic microalgae (genus: *Symbiodinium*), and a diverse internal and external microbial community, collectively forming the coral holobiont [2]. Although our understanding of the coral holobiont is still far from complete [3], we now recognize that the three partners in the association live and function in equilibrium [4] and that disruption of these interactions often leads to the breakdown of the symbiosis and death of the coral host.

Coral reefs have suffered massive reductions in abundance, diversity, and structure over the past 40 years [5, 6]. In 2008, a global assessment of reef health considered 19% of reefs degraded beyond repair, and identified 15 to 40% at severe risk of collapse [7]. Fast forward 9 years and two global mass mortality events later (in 2014 and 2016), and these projections now appear conservative. Aerial surveys of the Great Barrier Reef, revealed 90% of reefs in the northern section are showing signs of physiological stress [8]. A similar percentage of corals showed signs of thermal stress in the US Virgin Islands [9], while 75% of corals in Hawaii are considered to be at high risk [10]. The recent increases in the scale and global ubiquity of such losses has stimulated interest in understanding what determines coral health [3].

An increase in pathogen-driven disease is one means of disrupting the stability and functioning of the holobiont. Coral-pathogen interactions are often triggered by changes in environmental conditions [2], with above-ambient seawater temperatures known to be particularly important predictors of the prevalence and severity of coral disease outbreaks [11, 12]. Seasonal fluctuations in disease prevalence are not a novel phenomenon [9, 13]. In fact, it is well documented that a coral’s susceptibility to infection and the linear progression of tissue lesions in a given species depend on ambient light and temperature [14]. Small polyped corals have been reported to ingest and digest numerous bacterial species [15–17], yet the coral/pathogen interactions that occur during the infection process are largely unknown.

Efforts to resolve such interactions have been previously hampered by two factors: (i) the lack of a tractable coral-pathogen model system that can be manipulated in a controlled, repeatable manner and (ii) the complexity of imaging microscale interactions. The temperature-dependent relationship that exists between the reef-building coral *Pocillopora damicornis* and the pathogen *Vibrio coralliilyticus* has been advocated as a model system for understanding the dynamics of infection [18, 19]. This disease, first described by Ben-Haim and Rosenberg in 2002 [20], causes bleaching at temperatures between 24 °C and 27 °C, and tissue lysis at temperatures above 27 °C [21–23]. At higher temperatures, the disease progresses quickly, making it a perfect model for studying the progression of infection over short timescales. Gavish and co-workers (*in revision)* have recently developed the Microfluidic Coral Infection (MCI) experimental platform, which facilitates real time microscopic observations of the infection process and the development of disease symptoms (such as lesions, biofilms or tissue necrosis) at high spatial and temporal resolution. Here, we used the MCI to inoculate *P. damicornis* with ^15^N-labeled *V. coralliilyticus* and fix the corals at different time-points in the infection process. Isotopically-labeling the pathogens enabled us to subsequently track the pathogens and their breakdown products in situ using correlative TEM/NanoSIMS.

## Methods

### Collection and maintenance of the corals

A single *Pocillopora damicornis* colony was collected from a coral nursery located at ~ 8 m depth in the Gulf of Aqaba (Eilat, Israel) and transferred to an aquarium at the Interuniversity Institute for Marine Sciences (Eilat, Israel), where it was supplied with ambient flowing seawater (24 ± 2 °C) and natural light, shaded in order to mimic conditions experienced on the reef (i.e. 300–400 μmol photons m^− 2^ s^− 1^ at midday). The coral was fragmented into small pieces (5 × 5 mm) in April 2016 and left to recover for a week in the aquaria before being transported to the Weizmann Institute of Science (Rehovot, Israel). On arrival, the fragments were placed in a custom-built raceway chamber consisting of three separate channels, which were suspended above a temperature-controlled water reservoir. A submersible pump was added to the reservoir to circulate water between the two layers [24]. Separation of the two layers ensured that any water-loss by evaporation was minimal and thus stabilized salinity in the system. Photosynthesis-saturating light levels (150 μmol photons m^− 2^ s^− 1^) were provided by alternating blue and white LED strips, which were glued to a Plexiglas shelf positioned 10 cm above the glass raceway. The coral fragments were provided with conditions that matched those in Eilat (temperature: 25 ± 1 °C, pH: 8.1 ± 0.2, salinity: 40, light-dark cycle: 13.5 L/10.5 h D), for 1 week prior to the experiment to allow the fragments time to recover from any stress incurred during transportation. Experimental fragments were selected based on visual confirmation of health (i.e. skeleton covered by tissue, polyps extended and no paling of the coenosarc or excess mucus production). At this point, the temperature in the raceway was increased to 31 ± 1 °C for 3 d to prime the fragments for bacterial infection with *Vibrio coralliilyticus* [23].

### Preparation of the inoculum

The modified *V. coralliilyticus* strain (YB2), which contains a plasmid encoding for the T3 DsRed fluorescent protein [24] was grown overnight in ^15^N-labeled growth media containing: 5 g L^− 1 15^N 98% Celltone powder (Cambridge Isotope Laboratories Inc., Tewksbury, MA, USA), 2 g L^− 1^ glucose, and 50 μg mL^− 1^ kanamycin dissolved in filtered seawater (0.22 μm; FSW). 12 h incubation at 31 °C with gentle shaking (150 rpm), resulted in an inoculum density of ~ 10^8^ cells mL^− 1^ (estimated from flow cytometry counts). The bacterial suspension was centrifuged for 10 min at 3500 rpm. The supernatant was then discarded, replaced with an equivalent volume of FSW and vortexed, before it was returned to the incubator (31 °C, 0 rpm) for a further 4 h. This step, prior to inoculation, was crucial because it enhanced the secretion of zinc-metalloproteases, which are considered potent toxins in the infection process [22, 25, 26]. Importantly, this step did not reduce the ^15^N-labeling in the bacteria because the pathogens were already in the stationary phase and were thus, no longer dividing. Motile bacteria present in the supernatant, were collected immediately before the start of the experiment and transferred to sterile Corning® cell culture flasks (Sigma Aldrich, St. Louis, MI, USA).

### Inoculation in the Microfluidic Coral Infection (MCI) experimental platform

Inoculations were conducted in the state-of-the-art MCI system using specifically-designed microfluidics chambers that were constructed from polydimethylsiloxane (PDMS). A detailed explanation of the system and how the microfluidics chambers are fabricated is provided by Gavish et al. (*in revision*), but the resulting product is a microchip that measures 5 × 1.5 × 5 cm (L × W × H) and contains four 250 μL volume chambers. Each chamber has an inlet and outlet tube made of polyethylene (ø = 0.8 mm), the latter of which is connected to a peristaltic pump, enabling similar flow rates (2.6 ± 0.8 mL h^− 1^) to be attained in all of the chambers. The chamber is sealed with an ApopTag® Plastic cover slip and transferred to the temperature-controlled microscope stage of an inverted fluorescence microscope (Olympus IX81, Tokyo, Japan). Temperature (31 ± 0.5 °C) was monitored via a probe, which was inserted directly into the PDMS chip.

Fragments were placed in the system 4 h before inoculation to give them time to acclimate to the conditions on stage.

**Fig. 1:**
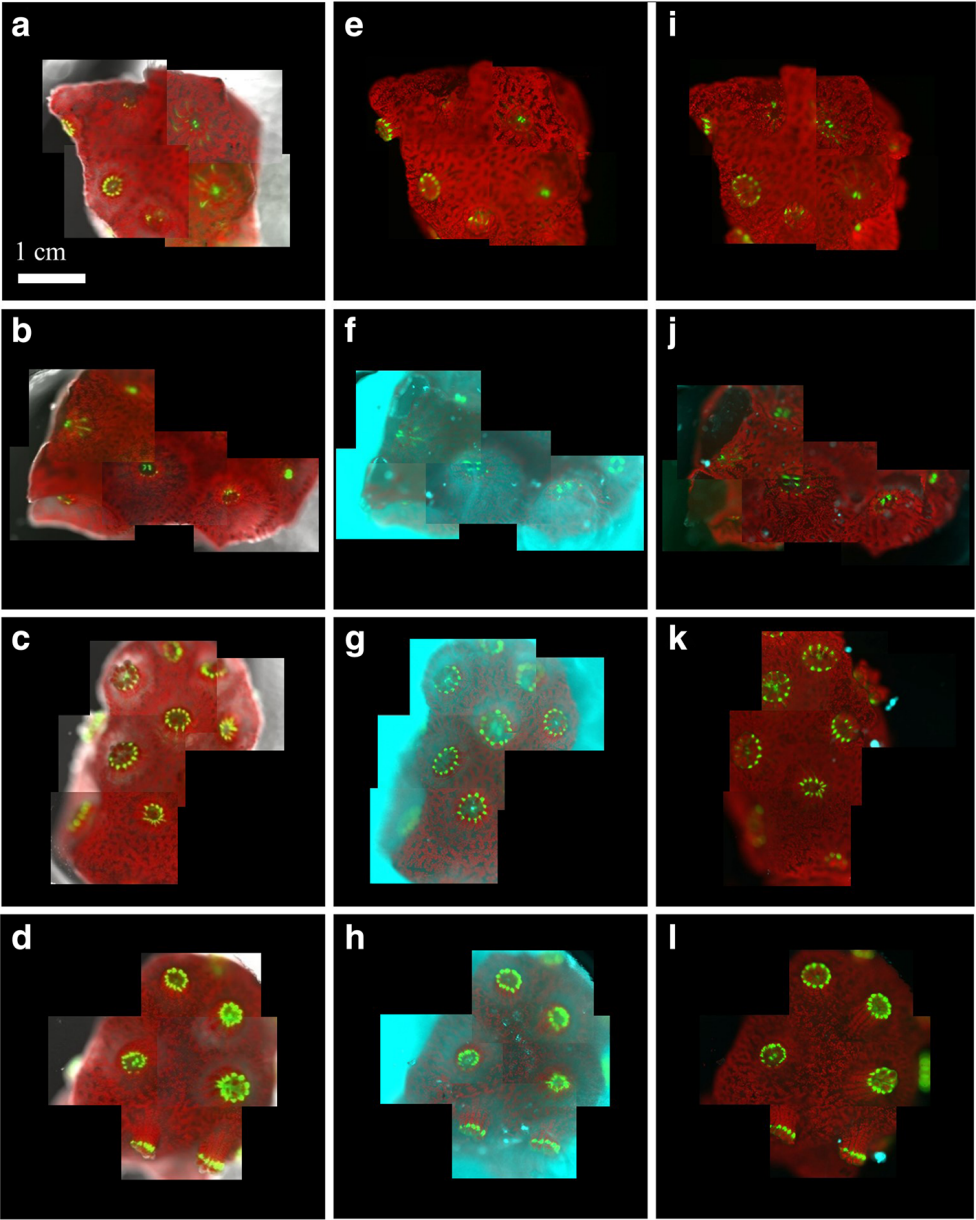
Live imaging of coral infection dynamics. *Pocillopora damicornis* fragments were placed in the Coral-on-a-Chip microfluidics system. One fragment was designated a control and was supplied with filtered seawater. The other three were inoculated with a modified *V. coralliilyticus* strain (YB2) for 2 h. Here we show: **a**-**d** the initial health of the four fragments before inoculation; **e**-**h** the state of fragment 1 h into the inoculation period and; **i**-**l** the state of the fragment at fixation. Corals were fixed (from top) at: 22 h (control), 2.5, 6, and 22 h *post*-inoculation. Fluorescence was captured in three channels: green fluorescent protein (Ex: 490 nm, Em: 535 ± 50 nm), chlorophyll (Ex: 490 nm, Em: 660 ± 50 nm), and DsRed (Ex: 555 ± 20 nm, Em: 590 ± 33 nm). See text for detailed explanation of the symptoms caused by disease

Images were taken of the coral fragments immediately before the inoculation period to confirm the health of fragments (Fig. 1a-d). Three of the four chambers were designated ‘infection chambers’ and were subsequently exposed to the 10^8^ cells mL^− 1^ inoculum, while the fourth chamber acted as a control and was exposed to FSW only. The inoculation period lasted 2 h. The inlet flow was then switched to FSW for the remaining incubation. Images were taken at four fixed positions on the coral surface, at 10 min intervals for the duration of the experiment using a Coolsnap HQ2 CCD camera (Photometrics, Tuscon, AZ, USA). Fluorescence was captured in three channels: green fluorescent protein (Ex: 490 nm, Em: 535 ± 50 nm), chlorophyll (Ex: 490 nm, Em: 660 ± 50 nm), and DsRed (Ex: 555 ± 20 nm, Em: 590 ± 33 nm). Between fluorescence imaging, the corals were provided with 250 μmol photons m^− 2^ s^− 1^ of white light, which was supplied by the microscopes transmitted light function. Because images were acquired in real time, we were able to visualize the progression of the infection and use the images to make a decision as to when to fix the samples (in 4% paraformaldehyde and 0.1% glutaraldehyde) for subsequent TEM/NanoSIMS imaging. Fragments were thus fixed at different stages of the infection process in line with the occurrence of symptoms of disease, assessed visually by the state of the tissue (confluence, coenosarc tearing, and polyp isolation).

### TEM and NanoSIMS imaging

Coral fragments were rinsed thoroughly in Sörensen sucrose phosphate buffer (0.1 M phosphate at pH 7.5, 0.6 M sucrose, 1 mM CaCl_2_) and decalcified in 0.5 M ethylenediaminetetraacetic acid (EDTA at pH 8) for 3 d at 4 °C. The remaining tissue was micro-dissected into single polyps using a binocular microscope. Polyps were post-fixed for 1 h in 1% osmium tetroxide, dissolved in distilled water. A series of washes (4 × 10 min) in distilled water followed, before the samples were dehydrated in a stepwise series of ethanol washes (3 × 10 min at 50, 70, 90, and 100%, respectively), and embedded in Spurr’s resin. One polyp *per* fragment was selected at random for processing and thin (70 nm) and semi-thin sections (500 nm) were cut using a 45° diamond knife (Diatome, Hatfield, PA, USA). Thin sections were stained with 4% uranyl acetate and Reynold’s lead citrate solution and imaged using a Philips CM 100 transmission electron microscope, located in the Electron Microscopy Facility (EMF) at the University of Lausanne (Switzerland). Initially we were unsure where the *V. coralliilyticus* would be localized and how abundant the pathogens would be in the tissue, so we created multiple high-resolution montages. These sections then were gold-coated and the same areas were imaged using a NanoSIMS 50 L ion microprobe. 

In the NanoSIMS, secondary ions were obtained by bombarding the sample with a beam of 16 keV Cs^+^ primary ions, focused to a spot-size of about 150 nm. The secondary ions ^14^N^12^C^−^ and ^15^N^12^C^−^ were counted in individual electron-multiplier detectors at a mass resolution power of about 9000 (Cameca definition), which is sufficient to resolve all potential interferences in the mass spectrum. Isotopic images (50 × 50 μm in size), were generated by rastering the primary beam across the surface of the sample, controlling the dwell time spent on each pixel (5 ms), the number of pixels (256 × 256), and the number of layers (5) for each image. Four tissues were analysed in each polyp: the oral epidermis, the oral gastrodermis, the aboral gastrodermis, and the mesenterial filaments (the majority of which consist of gastrodermis tissue; [27]). It was not possible to analyze the calicodermis, because this tissue layer was not preserved in sections. Between 5 and 14 images were obtained *per* tissue *per* coral fragment (*n* = 73 images in total). High-resolution images, typically 12 × 12 μm^2^, of specific, highly ^15^N-enriched sub-cellular structures were also obtained with a lateral resolution of ~ 100 nm. The software L’IMAGE (created by Dr. Larry Nittler, Carnegie Institution of Washington) was used to produce drift-corrected ^15^N-enrichment maps. All ^15^N-enrichment levels are expressed in the delta-notation:

$$ {\updelta}^{15}\mathrm{N}\ \left({\mbox{\fontencoding{U}\fontfamily{wasy}\selectfont\char104}} \right)=\left(\left({\mathrm{R}}_{\mathrm{sample}}/{\mathrm{R}}_{\mathrm{control}}\right)-1\right)\times 1000, $$

where R_sample_ is the ^15^N/^14^N ratio measured in the sample, and R_control_ is the measured ratio of a sample with natural ^15^N/^14^N ratio, prepared and analysed in an identical manner. For easy comparison, a scale from 0 to 4000 was applied to the δ^15^N (‰) images. This image, in conjunction with the ^12^C^14^N^−^ image, was used to draw regions of interest (ROI) around the tissue(s) present. The average δ^15^N (‰) was calculated for each tissue. The same method was used to define ROIs around ^15^N-hotspots (areas enriched above background levels) present in the tissues. We defined a “hotspot” as a ROI with a δ^15^N > 300 and a size > 10 pixels. The density of hotspots was subsequently calculated by dividing the number of hotspots by the area of tissue, and expressed as the number of hotspots *per* μm^2^.

### Statistical analysis

The tissue enrichment data was log-transformed to achieve normality (Kolgomorov-Smirnov, *p* > 0.05). The importance of time (ordinal factor: 2.5, 6, or 22) and tissue (nominal factor: oral epidermis, oral gastrodermis, aboral gastrodermis, and mesenterial filament) were analysed using a two-way analysis of variance (ANOVA). A Tukey’s honestly significant difference *post-hoc* test was used to identify where the differences lay in the event of a significant interaction being found. Analysing the hotspot density data was complicated by the number of images that contained zero hotspots (40 out of 111) and the high variability between images (which ranged from zero to 0.039 hotspots *per* μm^2^). The data could not be transformed to achieve normality and did not meet the criteria for the homogeneity of variance either, so a non-parametric Kruskal-Wallis test was used to compare structures at different time-points. In the event of a significant difference being found, a Nemanyi *post*-*hoc* test was used to identify where the differences lay.

## Results

### Live imaging of coral infection

All of the *P. damicornis* fragments were healthy before inoculation with *V. coralliilyticus*. Polyps were extended and no visible surface wounds were present (Fig. 1a-d). The control fragment, which was not exposed to the bacterial inoculum, remained healthy throughout the experiment (Fig. 1e, i). Infectivity differed markedly between the three fragments. One fragment experienced an extremely virulent infection, with two large lesions formed an hour into the inoculation period (Fig. 1f). The same fragment exhibited significant tearing of the coenosarc around one of the polyps and *V. coralliilyticus* were observed accumulating on the septa (Fig. 1j). This fragment was fixed at 2.5 h *post*-inoculation (hpi). The other infected fragments did not develop lesions, despite being exposed to the same inoculum and environmental conditions. Instead, the polyps became stretched and the coenosarc tissue lost confluence over time. We fixed one fragment midway through the light cycle at 6 hpi (Fig. 1k), and the other at the end of the dark period at 22 hpi (Fig. 1l). The control was also fixed after 22 h (Fig. 1i).

### Tracking ^15^N-labeled *V. coralliilyticus* in situ

**Fig. 2:**
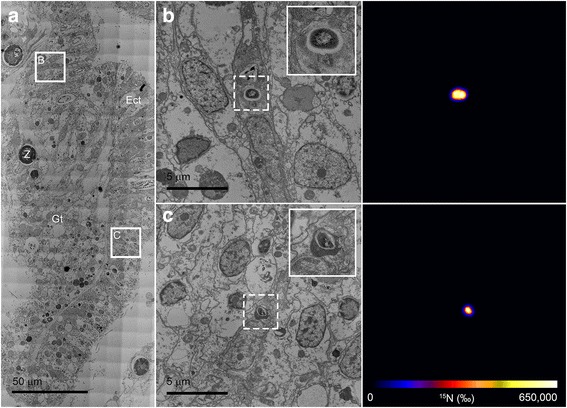
Localizing pathogens using correlative TEM/NanoSIMS. The reef-building coral *Pocillopora damicornis* was infected with ^15^N-labeled *Vibrio coralliitycus* and fixed at 2.5 h *post-*inoculation. **a** TEM montage of a representative coral tissue section consisting of oral epidermis (Ect), oral gastrodermis (Gt) and *Symbiodinium* cells (Z). **b** and **c** higher-resolution view of the squares labeled in (**a**). Each TEM image is pictured alongside its corresponding NanoSIMS ^15^N/^14^N image, which is scaled according to the isotopic enrichment in the sample (where blue represents natural ^15^N/^14^N enrichment levels of 0.0036 and white represents maximum enrichment)

Intact *V. coralliilyticus* were easily localised in inoculated *P. damicornis* fragments using the NanoSIMS because of their high ^15^N-enrichment; which was up to 650,000 ‰ (Fig. 2). The presence of *V. coralliilyticus* (which also contains a protein encoding for DsRed [24]) in inoculated polyps was further confirmed by immunolocalisation (Additional file 1). Single *V. coralliitycus* were observed in all tissue layers (oral epidermis, oral gastrodermis, aboral gastrodermis, and mesenterial filament) using both techniques (Fig. 2 and Additional file 1). Out of 73 NanoSIMS images that were taken, we were able to identify 14 ^15^N-labeled *V. coralliilyticus*. Of these, 11 were found in the fragment fixed at 2.5 hpi, one was found in the fragment fixed at 6 hpi and two were observed in the fragment fixed at 22 hpi. Nine of the 11 *V. coralliilyticus* cells observed in the fragment fixed at 2.5 hpi were located in the oral epidermis, one was in the mesenterial filaments, and one was in the aboral gastrodermis. The single *V. coralliilyticus* observed in the 6 hpi fragment was located in the oral gastrodermis, while the two observed in the 22 hpi fragment were located in the mesenterial filaments. *Vibrio coralliilyticus* were typically located in the columnar epithelial cells that dominate the oral epidermis (Fig. 2a). In some cases, the pathogens appeared intact (Fig. 2b); while in others the pathogens were enclosed within electron-dense intracellular vesicles (Fig. 2c).

### Tracking the ^15^N-labeled breakdown products of *V. coralliilyticus* in the coral polyp

**Fig. 3:**
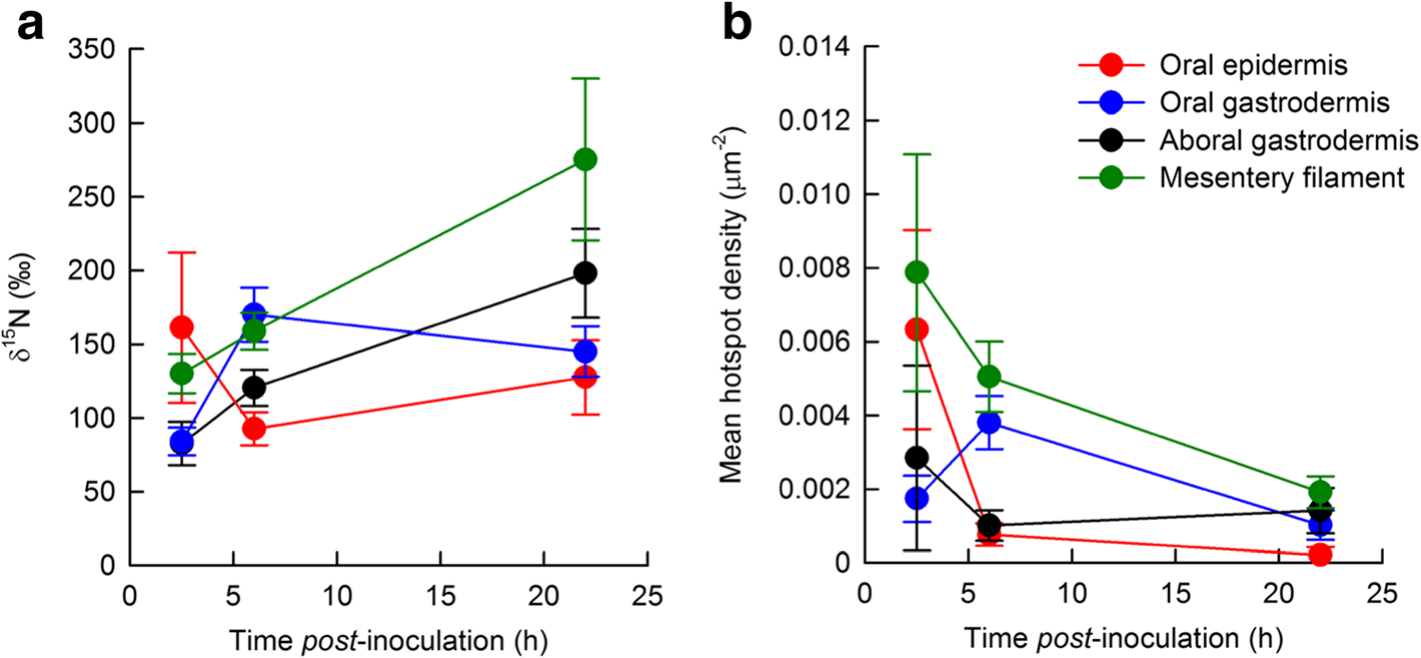
Identifying the major sites of metabolic activity during infection. *Pocillopora damicornis* was infected with ^15^N-labeled *Vibrio coralliitycus* and fixed at 2.5, 6 and 22 h *post-*inoculation. NanoSIMS images were taken of each polyp and a standardised scale (0 to 4000) was applied to the resulting ^15^N/^14^N images. The software L’IMAGE was used to draw regions of interest around tissue layers and ^15^N-hotspots (where δ^15^N > 300, size > 10 pixels). **a** Mean δ^15^N in four tissues: the oral epidermis (red), the oral gastrodermis (blue), the aboral gastrodermis (black), and the mesenterial filaments (green). **b** Mean hotspot density in the same four tissues, relative to the area of the tissue imaged. Values represent mean ± S.E.M, *n =* 5-14 images, *per* tissue *per* coral fragment (*n* = 73 images in total)

**Fig. 4:**
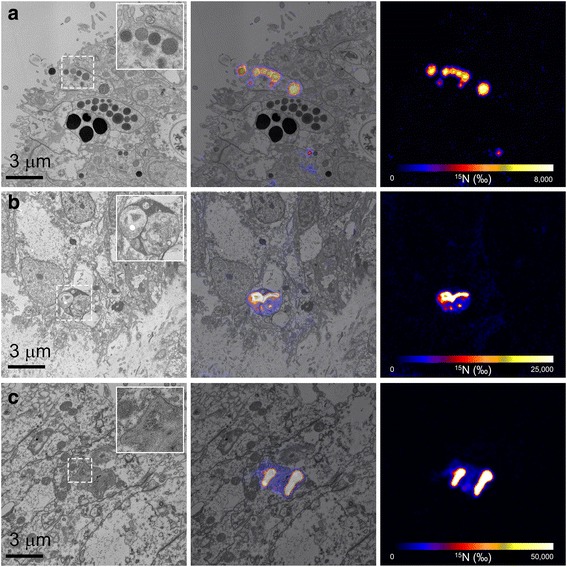
Highly-enriched (sub) cellular structures in the coral polyp. **a** Epidermal secretory cell extruding vesicles containing the degradation products of pathogens observed at 2.5 h *post*-inoculation. **b** and **c** Phagosomes located in the host mesenterial filaments observed at 6 h *post*-inoculation. Each TEM image is pictured alongside its corresponding NanoSIMS ^15^N/^14^N image, which is scaled according to the isotopic enrichment in the sample (where blue represents natural ^15^N/^14^N enrichment levels of 0.0036 and white represents maximum enrichment)

The distribution and density of *V. coralliilyticus*-derived products among different tissue layers is presented in Fig. 3, while representative, highly ^15^N-enriched intra-cellular structures are provided in Fig. 4. Original data and additional TEM/NanoSIMS montages are provided as Supplementary Information (Additional files 2, 3, 4, 5 and 6). The level of ^15^N-enrichment depended on both the time of fixation and the structure, resulting in a significant ‘time × structure’ interaction (F_(6, 99)_ = 2.280, *p* = 0.042). ^15^N-enrichment levels increased linearly over time in the mesenterial filaments, the oral gastrodermis, and the aboral gastrodermis. In contrast, ^15^N-enrichment in the oral epidermis (the most strongly enriched tissue at 2.5 hpi) decreased 43% between 2.5 and 6 hpi but then remained stable until 22 hpi (Fig. 3a). Interestingly, enrichment in the oral gastrodermis showed the opposite trend to the oral epidermis, with levels doubling between 2.5 and 6 hpi, before stabilizing between 6 and 22 hpi (Fig. 3a).

The density of ^15^N-hotspots was statistically comparable among tissue types at 2.5 hpi (Chi-square = 5.172, df = 3, *p* = 0.160). However, the density of hotspots significantly differed between structures at 6 hpi (Chi-square = 18.042, df = 3, *p* = < 0.001) and 22 hpi (Chi-square = 10.451, df = 3, *p* = 0.015). *Post*-hoc analyses revealed that ^15^N-enrichment was comparable between the mesenterial filaments and the oral gastrodermis, and between the aboral gastrodermis and the oral epidermis at 6 hpi (Fig. 3b), but that levels were 4 to 6-fold higher in the mesenterial filaments and oral gastrodermis, compared with the aboral gastrodermis and the oral epidermis. At 22 hpi, the only significant difference that was observed was between the oral epidermis and the mesenterial filaments (Fig. 3b).

Closer examination revealed that hotspots in the oral epidermis were typically restricted to secretory-type host cells (Fig. 4a), while in the mesenterial filaments they tended to co-localize with phagosomal structures (Fig. 4b, c). ^15^N-labeling was not uniform throughout these structures. Epithelial secretory-type cells contained both ^15^N-labeled secretory vesicles and granules (Fig 4a), in close proximity to labelled Golgi stacks. ^15^N-enrichment levels and patterns differed between phagosomes (Fig. 4b, c). It is possible that this heterogeneity reflects different stages of digestion, or possibly, different numbers of pathogens that are engulfed.

## Discussion

Recent increases in the scale and prevalence of coral disease [13] has increased the need to understand the causes and consequences of infection in these key ecosystem engineers. Questions concerning the immune capacity of a coral arise when challenged with pathogenic bacteria under elevated temperature. A major gap in our understanding of coral disease is the sequence of cellular events during infection. How do pathogens colonize their hosts and propagate in deeper tissues? The challenge posed by questions such as these is dichotomous with regards to scale: coral disease tends to be diagnosed in the field using macroscopic symptoms of disease such as lesions and/or loss of tissue, yet coral/pathogen interactions occur at microscopic (i.e., subcellular) scales. To date, examination of such interactions are few at the tissue level [22, 23, 26] and lacking at the (sub-)cellular level. We provide a new approach for studying coral-pathogen interactions at microscale resolution using a combination of stable isotopes, microfluidics, and NanoSIMS. By growing pathogenic bacteria in ^15^N-enriched media and inoculating our model coral *P. damicornis* at temperatures permissive to infection, we are able to visualize the penetration and dispersal of *V. coralliilyticus* (and their degradation or secretion products), at different stages of the infection process.

Infectivity differed greatly among the three fragments despite all the fragments being exposed to the same inoculum (~ 10^8^ *V. coralliilyticus* cells mL^− 1^). The first hour of inoculation induced identical responses in the infected fragments. Initial contact with the *V. coralliilyticus* caused the polyps to retract into their calices. Within 30 min, *V. coralliilyticus* began to accumulate in the polyp mouth region and after an hour, the coenosarc tissue started to become stretched. At this point, differences became evident in the responses of the three fragments. Two (those fixed at 6 and 22 hpi) began to spew pathogen-laden mucus from the mouths of their polyps and stretch their mesentary filaments across the surface of the coral, but the remaining fragment (fixed at 2.5 hpi) did not (Fig. 1). Instead, two large lesions, surrounded by *V. coralliilyticus* and sloughed-off mucus, began to form. These observations support the idea that host behavioural responses play an important role in determining the virulence and lethality of the infection (Gavish et al. *in revision*). They also go some way towards explaining the differences in the number of *V. coralliilyticus* observed in the tissues of the inoculated fragments. Out of the 14 *V. coralliilyticus* that we observed in the coral tissue, 11 were detected in the fragment that experienced the most severe infection (2.5 hpi), one *V. coralliilyticus* was detected in the fragment at 6 hpi and two were observed at 22 hpi. The general paucity of *V. coralliilyticus* that we imaged is likely to be an artefact of the limited tissue area covered by NanoSIMS imaging, rather than low-labelling efficiency because ^15^N-enrichment levels in *V. coralliilyticus* remained high, even in pathogens that were imaged at 22 hpi (up to 65,000 ‰). Future studies can overcome this artefact by combining our technique with methods such as immunolocalisation (protocol included in the Supplementary Information; Additional file 1), which are able to cover a much larger sampling area and depth.

Of the *V. coralliilyticus* that we did observe with the NanoSIMS, 9 out the 11 pathogens imaged in the fragment fixed at 2.5 hpi were found in the oral epidermis; generally, in columnar, epithelial-type cells (Fig. 2). This surface tissue layer, has previously been identified as being the most likely site of bacterial division [23], but has not been described as the point of entry of pathogens. The accumulation of the pathogenic *V. coralliilyticus* in the polyp pharynx that was observed by the live cell imaging here and by Gavish and co-workers (*in revision*), points towards a gastrovascular route of infection. The oral epidermis is lined by motile cilia, which beat continuously to increase flow at the surface of the coral and facilitate the entry of food into the coelenteron [28, 29]. In conditions that are permissive for infection, it is possible that these flows entrain pathogenic bacteria onto the coral surface, allowing contact prior to entry into the polyp. It is equally possible that the cilia provide defense against pathogen colonization by trapping pathogens in the surface mucus layer [30, 31]. Either way, the cilia are likely to play an important role in determining the outcome of coral-pathogen interactions [32].

Numerous studies have shown that microorganisms are actively or passively ingested by coral polyps [15, 33–35]. Prey is ingested via the stomodeum and the pharynx, with the mesenterial filaments playing important roles in both the ingestion and digestion of prey items. Thus, it was not surprising that the oral epidermis was not the only tissue layer in the polyp where *V. coralliilyticus* were observed. We also detected ^15^N-labeled *V. coralliilyticus* in the aboral gastrodermis (2.5 hpi), the oral gastrodermis (6 hpi), and the mesenterial filaments (2.5 hpi and 22 hpi). It is possible that these temporal differences reflect the path of *V. coralliilyticus* inside the coral polyp (moving from the oral epidermis to the oral gastrodermis including the mesenterial filaments, and then penetrating deeper into the coral polyp and into neighboring polyps via the gastrovascular cavity), although the low density of pathogens and low number of biological replicates precludes a definitive conclusion being reached on this hypothesis. Of note, we exclusively encountered single *V. coralliilyticus*; an observation that differs from previous studies, which described the formation of bacterial aggregates (known as bacteriocytes if they are enclosed in a host cell). This is likely to be a consequence of the shorter infection cycles used in our study. Bacteriocytes typically develop between 9 and 13 days [22, 23] after inoculation and tend to be associated with necrotic or severely degraded tissue. They are thus important indicators of the latter stages of infection, which were not reached in our experiment.

We also observed electron-dense intracellular vesicles enclosing the *V. coralliilyticus* cells (Fig. 2b). These are likely to be host-derived cell structures involved in the immune response [36]. Tissues of the mesentery filaments showed the highest hotspot density (regions of interest where δ^15^N > 300 and size > 10 pixels). These hotspots were particularly evident in the most heavily infected fragment (2.5 hpi), suggesting that these structures play an important role in the early stages of infection. If we compare the ^15^N-enrichment in the tissue with the number of hotspots present in the mesenteries we observe opposite responses. Levels of tissue ^15^N-enrichment increase over time, yet there is a reduction in the density of hotspots (Fig. 3). The dilution of the ^15^N signal into the surrounding tissue suggests that the turn-over of pathogen-derived material is faster in the mesenteries than in other tissue layers. The mesenteries are known to contain cell types and enzymes that are involved in the digestion of prey [35, 37]. They are also known to play an active role in cleaning the surface of the polyp [28]. Our results lead us to suggest that they may also play an important role in the digestion of bacterial pathogens during infection, acting not only in food digestion, but also in innate immunity.

Closer examination of the ^15^N-enriched hotspots imaged in the mesenteries revealed these tended to be dominated by phagosomal structures (Fig. 4b, c). In contrast, cells labelled in the oral epidermis were dominated by secretory-type cells (Fig. 4a). ^15^N-enrichment levels in the phagosomes were up to six times higher than the labelling in the secretory cells, suggesting phagosomes are the primary degradation site of pathogens [36] and that the nutrients are transferred to neighbouring cells. Interestingly, the secretory cells that contained highly ^15^N-enriched granules and labelled Golgi stacks (Fig. 4a), tended to be positioned close to the edge of the tissue, adjacent to the interface with the coelenteron. It is tempting from our observations, to propose that these two cell types form part of the rudimentary host immune system, with phagosomes breaking down the pathogen and transferring the detritus to secretory cells, which release the material into the surrounding seawater, although to affirm this, further studies are needed.

## Conclusions

In summary, we have presented here, a novel approach for assessing the dynamics of coral disease using stable isotope enrichment combined with microfluidics and correlative TEM/NanoSIMS imaging. In this *proof-of concept* study, we have proven that we are able to track ^15^N-labeled *V. coralliilyticus* and their breakdown products among tissue layers and into different cellular structures in the coral polyp. Our microscale approach has yielded several novel observations that would not have been possible using traditional techniques, which assess infection at the macroscale. Experimental replication and complementary techniques will be required to ascertain the route pathogens take once they are inside the polyp and to further define the immune capacity of the coral host. Our next step, will be to isotopically-label all three partners of the holobiont (using ^13^C-labeled seawater in combination with ^15^N-labeled pathogens) to determine how interactions between the partners (in terms of metabolic allocation) are altered in a disease scenario.

## Additional files

Additional file 1: Supplemental method for in situ immunolocalisation of *Vibrio coralliitycus* in coral tissue. (PDF 64 kb)

Additional file 2: Summary of the enrichment values generated from the NanoSIMS image analysis. (PDF 62 kb)

Additional file 3: Correlative TEM/NanoSIMS in the control fragment fixed at 22 h. (PDF 113 kb)

Additional file 4: Correlative TEM/NanoSIMS in the infected fragment fixed at 2.5 h. (PDF 189 kb)

Additional file 5: Correlative TEM/NanoSIMS in the infected fragment fixed at 6 h. (PDF 180 kb)

Additional file 6: Correlative TEM/NanoSIMS in the infected fragment fixed at 22 h. (PDF 170 kb)

## Abbreviations

EDTA: Ethylenediaminetetraacetic acid
FSW: Filtered seawater
HPI: Hours *post*-inoculation
MCI: Microfluidic coral infection
NanoSIMS: Nanoscale secondary ion mass spectrometry
PDMS: Polydimethylsiloxane
ROI: Regions of interest
TEM: Transmission electron microscopy
