# Supplementary material for: Using NanoSIMS coupled with microfluidics to visualize the early stages of coral infection by Vibrio coralliilyticus
Source: BMC Microbiol. 2018 Apr 20;18:39. doi: 10.1186/s12866-018-1173-0 (PMC5910561; doi:10.1186/s12866-018-1173-0)

Supplemental method for *in situ* immunolocalisation of *Vibrio coralliitycus* in coral tissue.

Individual coral polyps were orientated and embedded in Paraplast paraffin. Sections (5 µm) were cut using a LEICA microtome and de-paraffinised via a stepwise series of washes in 100% xylene (3×5 min) and 100% ethanol (3×5 min). For each replicate polyp, experimental sections and two negative controls (one control for the secondary antibody and one for auto-fluorescence), were mounted on same slide, delimited using a hydrophobic PAP pen. The tissues were rehydrated and permeabilised by a series of washes, using 0.2% triton dissolved in phosphate buffered saline (PBST, 1×10 min), 50 mM hydrochloric acid (HCl, 1×10 min), and PBST (3×3 min). A blocking solution consisting of 1% non-goat serum and 1% bovine serum albumin dissolved in PBST, was applied to prevent subsequent non-specific binding of the primary anti-body (1×30 min). Rat monoclonal anti-[5F8] red fluorescent protein (Chromotek) was the primary antibody used because it has high binding specificity to DsRed; the protein *V. coralliitycus* strain YB2 contains. Anti-RFP (diluted 1:1000 in blocking solution) was applied to the experimental sections and incubated in a humidity chamber (1×3 h). This and all subsequent steps were carried out in the dark at room temperature. After, the slide was rinsed in PBST (3×5 min), and the secondary antibody; anti-rat IgG (goat) conjugated to Alexa647 (Invitrogen) was applied at a 1:1000 dilution in blocking solution (1×1 h). Controls were: (i) sections exposed to secondary antibody without primary antibody, and (ii) no antibodies (to account for autofluorescence). Four further rinses were performed in PBST (2×5 min), PBS (1×5 min) and deionized water (1×5 min). The sections were then quickly rinsed in 90% ethanol and air-dried before mounting with ProLong Antifade mounting medium (Invitrogen). Fluorescence images were acquired with a PlanNeoFluar Zeiss 2.3X objective on a Zeiss Axiozoom V16, mounted with an Axiocam 506 camera. The microscope is located at the MNHN CeMIM platform (Paris, France). Bright field imaging was used to orientate in the coral tissue. Fluorescence imaging was conducted using structured light (Apotome mode of acquisition) using a Cy5 filter set (Ex 625-655 nm; Em 665-715 nm). Z-stack images (6 µm thick) were generated and reduced to 2D images using the Extended Depth of Focus algorithm. Below, is an example of the images generated. DsRed-tagged *V. coralliitycus* (YB2; bright red) were present in sections from infected polyps but not in the control polyps incubated in seawater. *Vibrios* were observed in the polyp mesenteries (mes.), the oral epidermis (ep), and both the oral and aboral gastrodermis (o. gastr., and ab. gastr.). The red autofluorescence of *Symbiodinium* cells is due to chlorophyll.

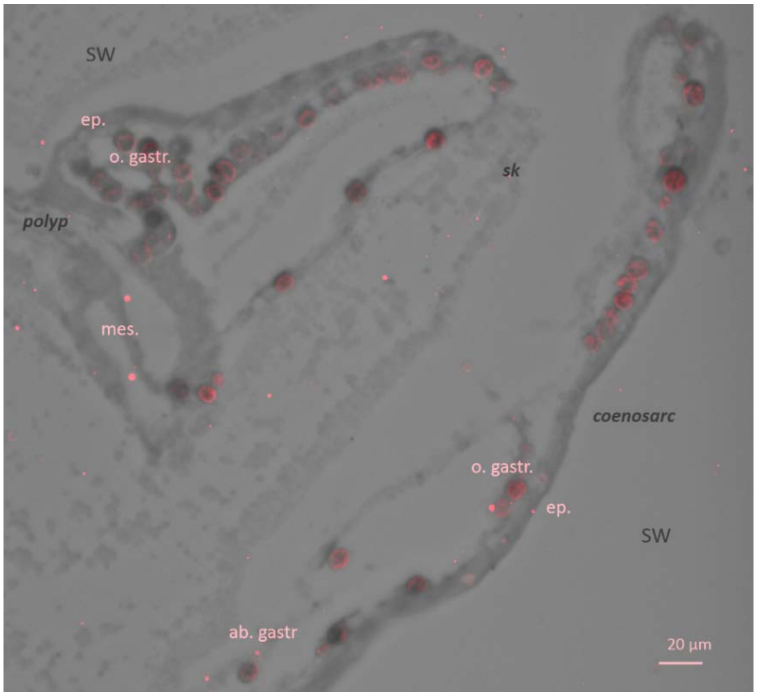

Supplement: Supplementary file 1 — Supplemental method for in situ immunolocalisation of Vibrio coralliitycus in coral tissue. (PDF 64 kb) [file 12866_2018_1173_MOESM1_ESM.pdf]
