# Supplementary material for: Using NanoSIMS coupled with microfluidics to visualize the early stages of coral infection by Vibrio coralliilyticus
Source: BMC Microbiol. 2018 Apr 20;18:39. doi: 10.1186/s12866-018-1173-0 (PMC5910561; doi:10.1186/s12866-018-1173-0)
Supplement: Supplementary file 2 — Summary of the enrichment values generated from the NanoSIMS image analysis. (PDF 62 kb) [file 12866_2018_1173_MOESM2_ESM.pdf]

Summary of the enrichment values generated from the NanoSIMS image analysis.

All values represent mean ± standard deviation and the number of replicate images are indicated.

Tissue	Time *post*-innoculation	Number of replicate images	Mean tissue $\delta^{15}N$ (‰)	Mean tissue area (μm^2)	Mean number of hotspots	Mean area of hotspots (μm^2)	Mean hotspot density (μm^2)
oral epidermis	control	9	1.575 ± 5.904	811.393 ± 575.817	-	-	-
	2.5	14	161.237 ± 190.741	994.854 ± 600.490	5.000 ± 6.409	7.549 ± 10.280	0.006 ± 0.010
	6	13	92.631 ± 40.441	650.938 ± 219.312	0.615 ± 0.870	0.517 ± 0.922	0.001 ± 0.001
	22	11	127.668 ± 83.703	921.614 ± 342.371	0.273 ± 0.905	0.670 ± 2.221	0.000 ± 0.001
oral gastrodermis	control	10	1.029 ± 6.021	1030.328 ± 835.437	-	-	-
	2.5	11	84.039 ± 31.318	1058.817 ± 565.785	1.545 ± 2.018	3.577 ± 5.633	0.002 ± 0.002
	6	14	170.018 ± 68.297	797.436 ± 535.837	3.429 ± 4.327	6.437 ± 6.175	0.004 ± 0.003
	22	9	145.000 ± 50.694	1124.131 ± 470.775	1.111 ± 1.269	1.518 ± 2.394	0.001 ± 0.001
aboral gastrodermis	control	6	1.039 ± 5.924	704.852 ± 288.324	-	-	-
	2.5	5	82.697 ± 32.714	787.275 ± 446.230	2.400 ± 4.336	4.916 ± 8.478	0.003 ± 0.006
	6	4	120.526 ± 24.546	1008.520 ± 396.564	1.250 ± 1.258	2.777 ± 3.810	0.001 ± 0.001
	22	10	198.049 ± 85.233	757.058 ± 194.009	1.000 ± 1.195	1.670 ± 2.091	0.001 ± 0.002
mesenterial filaments	control	7	-4.687 ± 5.397	2105.074 ± 379.954	-	-	-
	2.5	6	130.130 ± 32.806	1569.337 ± 370.752	10.500 ± 9.094	15.038 ± 14.655	0.008 ± 0.008
	6	7	158.797 ± 32.918	2187.853 ± 237.373	11.429 ± 6.655	29.416 ± 25.176	0.005 ± 0.003
	22	9	275.193 ± 164.475	1824.628 ± 392.891	3.778 ± 2.635	8.142 ± 6.418	0.002 ± 0.001
