# Supplementary material for: Using NanoSIMS coupled with microfluidics to visualize the early stages of coral infection by Vibrio coralliilyticus
Source: BMC Microbiol. 2018 Apr 20;18:39. doi: 10.1186/s12866-018-1173-0 (PMC5910561; doi:10.1186/s12866-018-1173-0)

Correlative TEM/NanoSIMS in the infected fragment fixed at 6 h PI.

Images taken in the mesentery filament (L-R): TEM image is pictured alongside its corresponding $^{15}N/^{14}N$ and $^{12}C^{14}N^{-}$ NanoSIMS images. The final column shows the TEM image overlaid with the $^{15}N/^{14}N$ image.

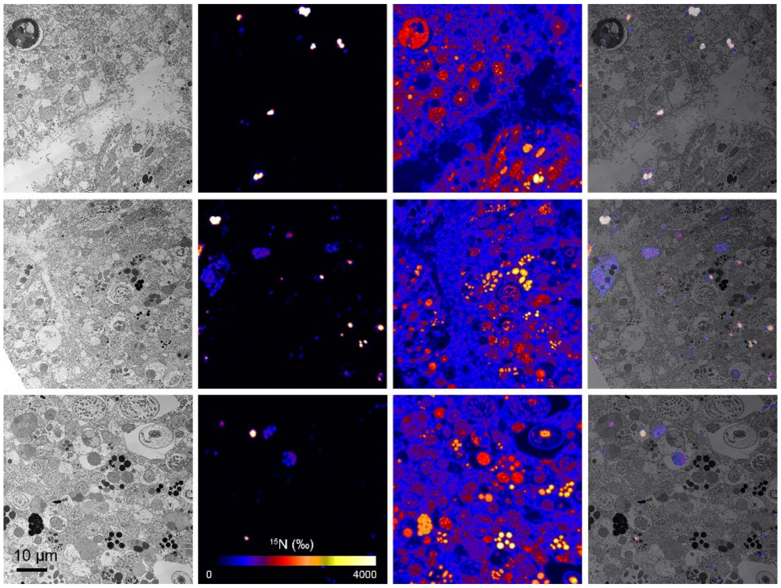

Supplement: Supplementary file 5 — Correlative TEM/NanoSIMS in the infected fragment fixed at 6 h. (PDF 180 kb) [file 12866_2018_1173_MOESM5_ESM.pdf]
